# Sociodemographic and Lifestyle Determinants of Plasma Oxidative Stress Markers 8-OHdG and F2-Isoprostanes and Associations with Metabolic Syndrome

**DOI:** 10.1155/2016/7530820

**Published:** 2016-02-23

**Authors:** Catherine N. Black, Mariska Bot, Peter G. Scheffer, Brenda W. J. H. Penninx

**Affiliations:** ^1^Department of Psychiatry and EMGO Institute for Health and Care Research, VU University Medical Center and GGZ inGeest, P.O. Box 74077, 1070 BB Amsterdam, Netherlands; ^2^Department of Clinical Chemistry, VU University Medical Center, Amsterdam, Netherlands

## Abstract

*Background.* Oxidative stress is increasingly important in health research. Therefore, it is necessary to understand which factors determine basal oxidative stress. This study examines the associations of various determinants with markers of oxidative DNA and lipid damage: 8-hydroxy-2′-deoxyguanosine (8-OHdG) and F2-isoprostanes.* Methods.* Data are from the Netherlands Study of Depression and Anxiety; 1117 subjects (18–65 years) without a current psychiatric diagnosis. Multivariable regression analyses were conducted with plasma levels of 8-OHdG and F2-isoprostanes (measured by LC/MS-MS) including sociodemographic, lifestyle, and sampling variables. Associations with metabolic syndrome (MetS) and chronic disease were examined.* Results.* 8-OHdG and F2-isoprostanes were weakly correlated (*r* = 0.06, *p* = 0.045). Both were positively associated with age and cotinine (cigarette exposure); 8-OHdG was lower in females and after longer sample storage. F2-isoprostanes were higher in females, alcohol users, and in samples collected in spring and lower in supplement users and those with more education. Both markers were lower in fasting subjects. F2-isoprostanes, not 8-OHdG, were positively associated with MetS.* Conclusion.* The weak correlation between 8-OHdG and F2-isoprostanes suggests they reflect specific aspects of oxidative stress. Both markers are associated with a range of sociodemographic, lifestyle, and sampling determinants which should be considered in future research. F2-isoprostanes are associated with MetS.

## 1. Introduction

The role of oxidative stress in physiological ageing is well-established and it is increasingly recognized as a key pathophysiological mechanism in a wide range of somatic diseases, including cancer, cardiovascular disease, and autoimmune, and neurological disorders, and more recently in psychiatric disorders and cognitive decline [1–3]. Oxidative stress, defined as an imbalance between reactive oxygen species (ROS; also known as free radicals) and antioxidant defences, can result in damage to lipids, proteins, and DNA leading to cellular dysfunction or ultimately cell death.

This study focuses on two widely used markers of oxidative damage: 8-hydroxy-2′-deoxyguanosine (8-OHdG) and F2-isoprostanes, reflecting oxidative DNA and lipid damage, respectively. 8-OHdG, first described in 1984 by Kasai et al. [4], is an oxidized derivate of the guanine base and one of the most abundant and extensively studied free radical induced DNA lesions. As a lesion with mutagenic potential, 8-OHdG has been studied especially in relation to carcinogenesis [5] and also in relation to occupational and environmental pollutants [6] and, among others, cardiovascular disease [7] and depression [8]. F2-isoprostanes are specific products of oxidation of the omega-6 fatty acid arachidonic acid and are chemically stable and measurable in all tissues and body fluids. F2-isoprostanes are currently argued to be the best available representative of oxidative lipid damage in the body as whole [9] and have been studied most extensively in the pathophysiology of cardiovascular disease [10].

Although these measurements of oxidative damage are widely used in human research and their clinical applicability is under investigation, knowledge of the factors that determine basal levels of 8-OHdG and F2-isoprostanes in the general population is incomplete. The majority of research on oxidative stress has been conducted in clinical samples with specific diseases or disorders.

Many previous studies have identified age, sex, and smoking status as determinants of oxidative stress [11–19], with higher levels of 8-OHdG and F2-isoprostanes in smokers as one of the most consistent findings. Associations with age and sex are more heterogeneous; F2-isoprostanes and 8-OHdG have been reported to be both higher [17, 20] and lower [14, 19] in females. 8-OHdG has been described to be both negatively [12] and positively [14] associated with age. Other frequently cited potential determinants include physical activity [21, 22], BMI [13, 23], and alcohol use [14, 18]. However, as studies have not always considered multiple covariates at the same time, it is hard to conclude which factors truly independently impact oxidative stress processes. Consequently, a comprehensive understanding of the main determinants of oxidative stress markers is not readily available. Furthermore, as oxidative stress is thought to be involved in the pathophysiology of most major chronic diseases, increased levels would be expected to be found in those subjects reporting current chronic diseases, or in those with known risk factors for the development of disease, such as the presence of (components of) metabolic syndrome (MetS). Previous reports on 8-OHdG and F2-isoprostanes in MetS have conflicting results; some find increased levels in subjects with MetS [24, 25], or associations with only some of the MetS components [14, 17, 26, 27], whereas others find no relationship [28–30].

Gaining insight into the determinants of these two important oxidative stress markers will allow a better understanding of the nature of these markers and help to identify potential confounding factors that need to be considered in future research. Therefore, this study investigates a wide range of sampling, sociodemographic, and lifestyle related determinants of plasma 8-OHdG and F2-isoprostanes in a large adult population sample. In addition, to explore whether 8-OHdG and F2-isoprostanes levels reflect health status the associations of both markers with the presence of self-reported chronic disease and (components of) metabolic syndrome are investigated.

## 2. Materials and Methods

### 2.1. Population

Data are derived from the baseline measurement of the Netherlands Study of Depression and Anxiety (NESDA), an ongoing longitudinal cohort study conducted among 2981 adults (aged 18 to 65 years) recruited from the general population, primary care, and mental health care organizations between September 2004 and February 2007 at three research sites in Netherlands. The study includes participants with and without psychiatric diagnoses. At baseline, participants in NESDA underwent a 4-hour assessment conducted by trained research staff according to a predesigned protocol, including blood withdrawal, written questionnaires, an interview and a physical examination. Depressive and anxiety disorders were ascertained using the lifetime version of the Composite International Diagnostic Interview (CIDI, version 2.1). A full, detailed description of the NESDA rationale, methods, and recruitment has been described in a previous publication [31].

To obtain an indication of the main determinants and associations with somatic health indicators of 8-OHdG and F2-isoprostanes unbiased by potential psychopathology and psychopharmacological effects, the present study only examined subjects without current psychiatric diagnoses or current antidepressant use. Therefore, 1814 of the 2981 subjects were excluded, leaving 1167 subjects. Of these, 16 subjects were excluded due to current pregnancy of breastfeeding, 34 had no data on both 8-OHdG and F2-isoprostanes, resulting in 1117 subjects (1114 with 8-OHdG measurements, 1025 with F2-isoprostane measurements). NESDA was approved by the Medical Ethics Committees of the participating institutes and all participants provided written informed consent.

### 2.2. Measurement of Plasma 8-OHdG and F2-Isoprostanes

Blood was collected the morning after an overnight fast using a Vacutainer blood collection tube and transported to local laboratory sites for processing within 1 hour of withdrawal. Plasma samples were stored at −80°C and transported to the Metabolic Laboratory of the VU University Medical Center where 8-OHdG and F2-isoprostanes were determined between 2012 and 2014.

#### 2.2.1. 8-Hydroxy-2′-Deoxyguanosine (8-OHdG)

Plasma levels of 8-hydroxy-2′-deoxyguanosine (8-OHdG) were determined by LC-MS/MS by adding 0.4 mL of 1 nmol/L internal standard (15N_5_ 8-OHdG, Buchem, Apeldoorn, Netherlands) in 3.4% phosphoric acid to 0.4 mL EDTA-plasma. The analytes were then extracted using Oasis mixed-mode anion exchange 96-well SPE plates (60 mg, Waters). Each well was successively washed with 1.5 mL 5% NH_4_OH, 1.5 mL 10% methanol, and 1.5 mL 100% methanol. The fraction containing 8-OHdG was eluted with 1 mL methanol containing 2% formic acid, dried under a steam of nitrogen at room temperature, and redissolved in 0.1 mL 5% methanol containing 0.1% acetic acid. A volume of 12 *μ*L was injected on a reverse phase HSS T3 column (1.8 *μ*m, 2.1 × 100 mm; Waters). The eluate components were separated at a flow rate of 0.45 mL/min using a gradient of Milli-Q water and methanol containing 0.1% acetic acid and were measured on an API 5000 mass spectrometer in positive ion multiple reaction monitoring acquisition mode. To calculate the 8-OHdG concentration, the analyte to internal standard peak area ratio with transitions 284.2 to 168.2 and 289.2 to 173.2, respectively, was compared with a standard curve up to 4.0 nmol/L 8-OHdG. Intra- and interassay CVs were 3.1% and 6.3%, respectively.

#### 2.2.2. F2-Isoprostanes

The total, that is, free and esterified, concentration of 8-iso-prostaglandin F2*α* (iPF2*α*-III) was determined by liquid chromatography tandem mass spectrometry (LC-MS/MS). In brief, 0.02 mL of 10 ng/mL deuterated internal standard (8iPF2*α*-d4; Cayman Chemical, USA) was added to 0.4 mL EDTA-plasma. To prevent arachidonic acid from autooxidation during sample preparation, butylated hydroxytoluene was added to a final concentration of 3 mmol/L. Then, 0.05 mL of 10 mol/L KOH and 0.4 mL MeOH were added for alkaline hydrolysis, and the samples were incubated in a shaking water bath for 60 min at 40°C. Afterwards, the samples were cleaned up using polymeric strong anion exchange 96-well solid-phase extraction (SPE) plates (Strata-X-A-33u 60 mg/well, Phenomenex, Torrance, USA). The wells were successively washed with 1 mL of 2% NH_4_OH, 1 mL hexane, and 1 mL 2-propanol using a positive pressure-96 processor (Waters, Milford, USA). The fraction containing F2-isoprostanes was eluted with 1 mL of 0.5% acetic acid in 2-propanol and then dried under a stream of nitrogen at room temperature and redissolved in 0.1 mL 25% acetonitrile containing 0.05% acetic acid. A volume of 15 *μ*L was injected on an Acquity BEH C18 column (Waters; 1.7 *μ*m, 2.1 × 100 mm). F2-isoprostanes were quantified by an API 5000 triple quadrupole mass spectrometer (AB Sciex Technologies, Toronto, Canada) in negative ion multiple reaction monitoring acquisition mode. To calculate the iPF2*α*-III concentration, the analytes to internal standard peak area ratio with transitions 353.2 to 193.2 and 357.2 to 197.2, respectively, were compared with a standard curve up to 5.6 nmol/L iPF2*α*-III (Cayman Chemical). The intra- and interassay variations were 4.6% and 8.2%, respectively.

### 2.3. Covariates

#### 2.3.1. Sociodemographics

These included sex (male/female), age (years), ethnicity (North European Ancestry, yes/no), and number of years of education.

#### 2.3.2. Lifestyle

Smoking status was self-reported and classified as nonsmokers [never and former], current smokers <10 cigarettes per day, and current smokers ≥10 cigarettes per day. Cotinine (the major metabolite of nicotine) levels were included as an additional measure of cigarette smoke exposure. Cotinine concentrations were assessed in blood plasma by solid-phase competitive ELISA (Cotinine Direct ELISA kit, Cat. number CO096D, Calbiotech, CA, USA). The detection limit was 1 ng/mL. Intra- and interassay coefficients of variation for values >2 ng/mL were <20% and <15%, respectively. Participants with cotinine values below the detection limit of 1 ng/mL had their level set at the value of 0.9 ng/mL.

Alcohol use was categorized based on units per week into mild/abstainer (<1 unit per week for men and women), moderate users (≤21 units per week for men, ≤14 units per week for women), and heavy users (>21 units per week for men, >14 units per week for women). Weight and height were measured and used to calculate Body Mass Index (BMI kg/m^2^). Physical activity (PA) was assessed using the 7-item International Physical Activity Questionnaire (IPAQ) [32] covering vigorous activity, moderate walking, and sitting activities over the past 7 days. PA and was expressed as a continuous measure of total MET (Metabolic Equivalent of Task) minutes per week, which is a ratio of the amount of energy expenditure during an activity to the expenditure at rest (http://www.ipaq.ki.se/).

The use of supplements was based on self-reported use in the month before sample collection and classified according to the Anatomical Therapeutic Chemical (ATC) classification [33]. Supplement use (yes/no) was defined as frequent use (more than half the days in the last month) of any or more of the following supplements: vitamin A (ATC A11CA01), vitamin E (ATC code A11HA03), vitamin C (ATC A11GA01), or a multivitamin supplement (ATC A11BA).

#### 2.3.3. Sampling Factors

Sampling factors included adherence to the instructions for overnight fasting prior to the exam (yes/no) and the season of sample collection (spring [March–May], summer [June–August], autumn [September–November], and winter [December–February]).

### 2.4. Metabolic Syndrome and Chronic Disease

The presence of metabolic syndrome (following the US National Cholesterol Education Program [NCEP], Adult Treatment Panel III [ATP III]) was defined as scoring above the established cut-off [34] in three or more of the following components: waist circumference (cm), triglycerides (mmol/L), high-density lipoprotein- (HDL-) cholesterol (mmol/L), systolic blood pressure (SBP) (mmHg), and fasting plasma glucose (mmol/L). In addition, a count variable was created reflecting the number of metabolic syndrome components in which a subject scored above the cut-off (range 0–5).

The metabolic syndrome components, as well as low-density lipoprotein (LDL) cholesterol (mmol/L) and diastolic blood pressure (DBP) (mmHg), were also analysed as continuous variables adjusted for medication use, by adding or subtracting mean changes after treatment. In subjects using fibrates, 0.10 mmol/liter was subtracted from HDL-cholesterol and 0.67 mmol/liter was added to triglycerides. In subjects using nicotinic acid, 0.15 mmol/liter was subtracted from HDL-cholesterol, and 0.19 mmol/liter was added to triglycerides. In subjects using any lipid-modifying agents 0.74 mmol/was added to LDL-cholesterol. In subjects using antihypertensive medication, 10 mmHg was added to the SBP and 5 mmHg to DBP. In subjects using antidiabetic medication, plasma glucose levels <7 mmol/L were set to 7 mmol/L.

As a global health indicator, a count of the number of chronic diseases was utilized and based on the self-reported presence of the following chronic diseases: asthma, chronic bronchitis or pulmonary emphysema, heart disease or myocardial infarction, diabetes, stroke, arthritis or arthrosis, rheumatic disorders, malignancies, high blood pressure, stomach or intestinal ulcers, other intestinal disorders, liver disease or liver cirrhosis, epilepsy, chronic fatigue syndrome, allergies, thyroid gland disease, injury (last year), head injury (ever), and other chronic diseases. In addition, to examine potential specificity of associations, we also examined the presence of the four most prevalent categories of major chronic disease clusters separately, including cardiometabolic disease (cardiovascular disease and/or diabetes), musculoskeletal disease (osteoarthritis and/or rheumatic disorders), chronic nonspecific lung disease, and cancer.

### 2.5. Statistical Analyses

Statistical analyses were conducted with SPSS version 20.0. A *p* value < 0.05 was considered statistically significant. 8-OHdG and F2-isoprostanes were log transformed to obtain a normal distribution for analysis. All analyses described were adjusted for research site. The association between 8-OHdG and F2-isoprostanes was examined in a linear regression analysis with 8-OHdG as independent and F2-isoprostanes as dependent variable.

Associations of 8-OHdG and F2-isoprostanes with the sociodemographic, lifestyle, and sampling variables were first examined individually in univariate linear regression analyses. Multivariable linear regression analyses were then conducted with 8-OHdG or F2-isoprostanes as dependent variables and all sociodemographic, lifestyle, and sampling variables in one model. The variance in oxidative stress levels explained by the determinants was reported as adjusted *R* squared.

Secondly, multivariable linear regression analyses were conducted with 8-OHdG and F2-isoprostanes as dependent variables and metabolic syndrome (components) or chronic disease as main predictors adjusted for the determinants identified in the previous step. In these analyses determinants with a *p* < 0.10 were included to minimize the chance of missing any relevant confounding variables.

## 3. Results

The mean age of the subjects in study sample was 42.5 years (SD = 14.1); 64.7% were female and had an average education of 12.7 (SD = 3.2) years (see Table 1). The mean 8-OHdG level was 45.5 pmol/L (SD = 17.0). Mean F2-isoprostane level was 120.6 pmol/L (SD = 40.4). 8-OHdG and F2-isoprostanes were weakly positively associated (*β* = 0.06, *p* = 0.045).

### 3.1. Sociodemographic, Lifestyle, and Sampling Factors 

#### 3.1.1. 8-OHdG

8-OHdG was higher with age (*β* = 0.16, *p* < 0.001) and lower in females (*β* = −0.14, *p* < 0.001).

8-OHdG was positively associated with both smoking and cotinine levels. In the multivariable model including both variables, only the association with cotinine levels remained significant (*β* = 0.09, *p* = 0.034). No association was found between 8-OHdG and alcohol use, physical activity, BMI, or supplement use. There was no association with the season of sample collection. Lower levels of 8-OHdG were found in subjects who had adhered to the fasting instructions (*β* = −0.06, *p* = 0.034; see Table 2). The explained variance of 8-OHdG levels by all determinants, expressed as adjusted *R* squared, was 0.052.

#### 3.1.2. F2-Isoprostanes

F2-isoprostanes were higher with age (*β* = 0.07, *p* = 0.043) and in females (*β* = 0.15, *p* < 0.001) and lower in those with more years of education (*β* = −0.10, *p* = 0.001). Plasma cotinine levels were associated with F2-isoprostanes (*β* = 0.09, *p* = 0.027), but smoking status was not. F2-isoprostanes were higher in heavy alcohol users (*β* = 0.13, *p* < 0.001) and lower in supplement users (*β* = −0.06, *p* = 0.020). No association was found between F2-isoprostanes and physical activity or BMI. Samples collected in spring or summer versus winter had higher levels of F2-isoprostanes (*β* = 0.11, *p* = 0.002; *β* = 0.07, *p* = 0.043). F2-isoprostanes were lower in those who had adhered to the fasting instructions (*β* = −0.07, *p* = 0.014). The explained variance of F2-isoprostanes levels by all determinants, expressed as adjusted *R* squared, was 0.060.

### 3.2. Metabolic Syndrome and Chronic Disease

#### 3.2.1. 8-OHdG

8-OHdG was not associated with any of the metabolic parameters or with the presence of metabolic syndrome (see Table 3). No associations were found with the number of chronic diseases (*β* = 0.02, *p* = 0.589) or the chronic disease categories (all *p* > 0.05).

#### 3.2.2. F2-Isoprostanes

F2-isoprostanes were higher with greater waist circumference (*β* = 0.08, *p* = 0.026), higher triglycerides (*β* = 0.21, *p* < 0.001), higher HDL-cholesterol (*β* = 0.23, *p* < 0.001), higher LDL-cholesterol (*β* = 0.19, *p* < 0.001), higher systolic (*β* = 0.13, *p* < 0.001), and diastolic (*β* = 0.13, *p* < 0.001) blood pressure. No association was found with fasting glucose (*β* = 0.02, *p* = 0.704). F2-isoprostanes were associated with both the presence of metabolic syndrome (*β* = 0.08, *p* = 0.013) and the number of metabolic syndrome components (*β* = 0.11, *p* = 0.001). No associations were found with the number of chronic diseases (*β* = 0.00, *p* = 0.998) or the chronic disease categories (all *p* > 0.05).

## 4. Discussion

This study examined sociodemographic, lifestyle, and sampling determinants of two plasma markers of oxidative damage 8-OHdG and F2-isoprostanes and their relationship with self-reported chronic disease and metabolic syndrome as health indicators in a large adult sample. 8-OHdG and F2-isoprostane plasma levels were found to be only weakly correlated suggesting that they largely reflect differential oxidative processes. Age, sex, level of education, plasma cotinine levels, alcohol consumption, supplement use, fasting prior to sample collection, and the season of sample collection were identified as determinants of either 8-OHdG or F2-isoprostanes, or both. No relationship with either marker was found for North European (versus any other) ancestry, BMI, or physical activity. In analyses corrected for these factors, F2-isoprostanes, but not 8-OHdG, were positively associated with all components of metabolic syndrome, except fasting glucose.

Previous studies that investigated the correlation between these two markers have mixed results. No correlation was found between plasma F2-isoprostanes and leucocyte 8-OHdG [35], nor between urinary F2-isoprostanes and 8-OHdG in lymphocytes [36]. The fact that in these studies levels were being compared across different biological samples may explain the lack of a relationship. However, studies comparing both markers across urine samples do not have consistent findings either and reported strong [37], moderate [16, 38], and absent [14] correlations. This study's finding of significant but very weak association between plasma 8-OHdG and F2-isoprostanes supports the suggestion that these markers reflect distinct pathways of oxidative damage that are not necessarily closely interrelated. The weak association also suggests that findings for one oxidative marker cannot be directly translated to another marker and confirms the importance of measuring multiple markers of oxidative stress.

### 4.1. Determinants

Both plasma 8-OHdG and F2-isoprostanes were positively associated with age, suggesting the accumulation of oxidative damage with the increasing age. This lends support to the hypothesis that oxidative damage accumulates with time and so contributes to ageing and age-related disease [39]. Female sex has previously been associated with lower 8-OHdG levels [11, 18] as was also found in the present study. However, others have reported no differences in 8-OHdG between males and females [14, 16, 36, 38, 40]. F2-isoprostanes have been demonstrated to be higher in females compared to males in both plasma [20, 24, 26] and urinary samples [17, 38] in agreement with this study's findings. The direction of the association between F2-isoprostanes and sex, however, is not consistent; another large-scale population sample demonstrated slightly lower levels of urinary F2-isoprostanes in women than men [19]. It has been hypothesized that differences in body composition between male and females and the influences of sex hormones in (premenopausal) females underlie the sex differences in oxidative stress levels. Although it is clear that sex is an important determinant of oxidative stress, the reasons for the differences between males and females, and the inconsistencies in the direction of the findings across studies, remain to be explored.

The inverse relationship of F2-isoprostanes with years of education as an indication of socioeconomic status (SES) has previously been described in another large-scale population sample, where lower SES by education, occupation, and income was associated with higher plasma F2-isoprostanes [41]. This may be a reflection of unmeasured environmental or occupational factors that accompany lower socioeconomic status and are associated to increased exposure to ROS.

In this study, self-reported smoking status was associated with 8-OHdG, but not with F2-isoprostanes. Higher levels of urinary 8-OHdG in smokers have been reported previously [11, 12, 18, 42]. In the multivariable analyses, 8-OHdG and smoking status remained significantly associated if cotinine was not included in the model (data not shown). The lack of an association between smoking status and F2-isoprostanes was unexpected, as higher F2-isoprostanes in smokers have frequently been reported in both urine [14, 17, 38, 42] and plasma samples [26, 42]. However, one study [20] found* lower* plasma levels of F2-isoprostanes in smokers in a sample of 298 healthy adults. A possible explanation for the lack of an association between smoking and F2-isoprostanes in this study may be the relatively low number of very heavy smokers, with only 3.1% of the sample smoking more than 20 cigarettes per day. F2-isoprostanes were however associated with plasma cotinine levels, which are arguably a more valid and sensitive measure of cigarette smoke exposure than self-reported smoking status [43], confirming exposure to cigarette smoke is associated with increased F2-isoprostane formation.

Ingestion of alcohol by healthy individuals has been demonstrated to directly increase urinary F2-isoprostane levels in [44] and a reduction of plasma F2-isoprostanes levels has been documented following the reduction of alcohol intake in moderate to heavy drinkers [45]. In this study, elevated F2-isoprostanes were associated with alcohol use, however only in those defined as heavy users, suggesting that moderate alcohol use is not associated with increased oxidative damage. The lack of an association in this study of either marker with BMI may be attributed to the relatively narrow range of BMI in this sample, especially in comparison with studies from the United States. The association with BMI, however, showed a trend towards significance in F2-isoprostanes, and F2-isoprostanes were found to be positively associated with waist circumference.

The significant influence of the season of sample collection found for F2-isoprostanes, with higher levels in spring and summer, may be a reflection of the impact of more sunlight and therefore UV radiation exposure that is present in Northwestern Europe in these seasons.

### 4.2. Metabolic Syndrome and Chronic Disease

Higher plasma F2-isoprostanes have been repeatedly demonstrated in subjects with CVD [46] and increased levels are also present in the early stages of atherosclerosis measured by coronary artery calcification (CAC) [26]. This study's results confirm that plasma F2-isoprostanes are also associated with most of the well-established risk factors for the development of atherosclerosis, including waist circumference, systolic blood pressure, triglycerides, and LDL-cholesterol levels, independent of sociodemographic and lifestyle factors. A previous study (*N* = 294) reported that plasma F2-isoprostanes were associated with waist circumference, systolic blood pressure, and presence of MetS [24]. An unexpected finding was the positive association with HDL-cholesterol levels. As low HDL-cholesterol levels are a well-established risk factor for CVD, an association in the opposite direction would be expected. This is however not the first study to demonstrate this association; plasma F2-isoprostanes were also positively associated with HDL-cholesterol in a population sample containing both healthy subjects (*N* = 227) and those with self-reported history of CVD (*N* = 440) [47].

Higher 8-OHdG levels have also been demonstrated in subjects with CVD and heart failure [7] and 8-OHdG has been found to be increased in atherosclerotic plaques [48]. This study did not find an association between plasma 8-OHdG and any of the metabolic syndrome components. A previous smaller study (*N* = 90) on the association between urinary 8-OHdG and metabolic syndrome components in healthy adults also found no association with metabolic syndrome components, with the exception of triglycerides [27].

Studies of clinically selected samples have found increased oxidative stress levels in persons with a wide range of chronic diseases, including cardiovascular disease, cancer, chronic obstructive lung disease, and musculoskeletal disorders, including osteo- and rheumatoid arthritis [49, 50]. The absence of any association between the chronic diseases and either 8-OHdG or F2-isoprostanes in this study is likely due to the relatively young age in this sample, the low prevalence of chronic diseases, and the limited specificity of self-reported measures.

### 4.3. Strengths and Limitations

The main strengths of this study are the sample size, the use of LC/MS-MS for the determination of two markers of oxidative damage in a sample with a well-documented, and wide range of potential determinants. As this is a cross-sectional study, the results cannot be interpreted as demonstrating causal relationships. A limitation of this study is that the effect of dietary patterns was not investigated.

### 4.4. Summary and Conclusion

In summary, plasma 8-OHdG and F2-isoprostanes levels are only weakly associated implying that they reflect specific aspects of oxidative damage. They are associated with a range of overlapping and differing sociodemographic, lifestyle, and sampling determinants which deserve consideration in future oxidative stress research. F2-isoprostanes, but not 8-OHdG, are associated with most components of metabolic syndrome, confirming their role in the pathophysiology of cardiovascular disease.

## Figures and Tables

**Table 1:** Sample and subject characteristics (*N* = 1117) of participants with plasma 8-OHdG and/or F2-isoprostane measurement.

	Mean (SD) or %
Sociodemographics	
Age (years)	42.5 (14.1)
Female	64.7%
Education (years)	12.7 (3.2)
North-European ancestry	96.6%

Lifestyle	
Smoking	
None (never/former)	69.3%
<10 cig/day	14.8%
≥10 cig/day	15.9%
Plasma cotinine ng/mL	71.2 (175.9)
Alcohol	
♂/♀ < 1 units/wk	23.2%
♂ ≤ 21/♀ ≤ 14 units/wk	65.0%
♂ > 21/♀ > 14 units/wk	11.8%
Physical activity (MET-min/wk)	3742 (3072)
Body mass index (BMI) (kg/m^2^)	25.3 (4.5)
Supplement users	11.5%

Metabolic, health, and disease factors	
Waist circumference (cm)	88.5 (13.4)
Triglycerides (mmol/liter)	1.3 (0.9)
HDL-cholesterol (mmol/liter)	1.6 (0.4)
LDL-cholesterol (mmol/liter)	3.1 (1.0)
Fasting glucose (mmol/liter)	5.2 (0.9)
Systolic blood pressure (mmHg)	137 (20)
Diastolic blood pressure (mmHg)	81 (11)
Metabolic syndrome (MetS)	19.6%
*N* of MetS components	1.4 (1.3)
*N* chronic diseases	
0	62.3%
1	26.6%
2 or more	12.1%
Cardiometabolic (cardiovascular disease, diabetes)	8.7%
Musculoskeletal (osteoarthritis, rheumatic disorders)	9.4%
Chronic nonspecific lung disease	6.5%
Cancer	3.3%

Sampling variables	
Season at time of sample collection	
Winter (Dec.–Feb.)	20.3%
Spring (Mar.–May)	24.5%
Summer (Jun.–Aug.)	24.5%
Autumn (Sep.–Nov.)	30.6%
Fasting prior to sample collection	95.6%

Oxidative stress markers	
8-Hydroxy-2′-deoxyguanosine (8-OHdG) (pmol/liter)	45.5 (17.0)
F2-isoprostanes (pmol/liter)	120.6 (40.4)

Cig: cigarettes; HDL: high-density lipoprotein; LDL: low-density lipoprotein; *N*: number; MET: metabolic equivalent of task; MetS: metabolic syndrome; Wk: week.

**Table 2:** Associations of sociodemographic, lifestyle, and sampling factors with plasma 8-OHdG and F2-isoprostanes.

	8-OHdG				F2-isoprostanes			
	Univariate		Multivariable		Univariate		Multivariable	
	*N* = 1114		*N* = 1104		*N* = 1025		*N* = 1017	
	*β*	*p*	*β*	*p*	*β*	*p*	*β*	*p*
Sociodemographics								
Age (years)	0.16	<0.001	0.16	<0.001	0.07	0.018	0.07	0.043
Female sex (male is ref.)	−0.16	<0.001	−0.14	<0.001	0.13	<0.001	0.15	<0.001
Education (years)	−0.04	0.154	−0.02	0.558	−0.09	0.002	−0.10	0.001
North-European ancestry (“NO” is ref.)	0.05	0.128	0.05	0.129	0.00	0.915	−0.01	0.738

Lifestyle								
Smoking								
Ref. nonsmokers	Ref.		Ref.		Ref.		Ref.	
<10 cig/day	0.08	0.010	0.05	0.120	0.04	0.191	0.02	0.546
≥10 cig/day	0.11	<0.001	0.05	0.398	0.02	0.497	−0.07	0.098
Plasma cotinine (ng/mL)	0.12	<0.001	0.09	0.034	0.07	0.013	0.09	0.027
Alcohol								
ref. < 1 unit/wk	Ref.		Ref.		Ref.		Ref.	
♂ ≤ 21/♀ ≤ 14 units/wk	0.04	0.233	0.00	0.921	0.01	0.818	0.06	0.094
♂ > 21/♀ > 14 units/wk	0.03	0.326	−0.02	0.553	0.12	<0.001	0.13	<0.001
Body mass index (kg/m^2^)	0.04	0.160	−0.02	0.594	0.06	0.053	0.05	0.099
Physical activity (MET-min/wk)	0.00	0.925	0.00	0.926	0.00	0.882	−0.02	0.440
Supplement use (“NON user” is ref.)	0.02	0.456	0.01	0.848	−0.05	0.076	−0.07	0.020

Sampling variables								
Season of sample collection								
Ref. winter (Dec.–Feb.)	Ref.		Ref.		Ref.		Ref.	
Spring (Mar.–May)	0.00	0.997	0.02	0.674	0.10	0.005	0.11	0.002
Summer (Jun.–Aug.)	−0.02	0.606	0.01	0.884	0.05	0.153	0.07	0.043
Autumn (Sep.–Nov.)	−0.01	0.877	0.01	0.729	−0.03	0.493	−0.01	0.776
Fasting prior to sample collection (“NO” is ref.)	−0.08	0.010	−0.06	0.034	−0.06	0.050	−0.07	0.014

8-OHdG and F2-isoprostanes were log transformed for analysis. All analyses are adjusted for research site. Results are reported as standardized regression coefficients. Cig: cigarettes; kg: kilogram; m^2^: meters squared; MET: metabolic equivalent of task; min: minutes; ref.: reference; Wk: week.

**Table 3:** Associations of metabolic factors and chronic disease with plasma 8-OHdG and F2-isoprostanes.

	8-OHdG^a^		F2-isoprostanes^b^	
	*β*	*p*	*β*	*p*
Waist circumference (cm)	−0.06	0.071	0.08	0.026
Triglycerides^c^ (mmol/L)	−0.04	0.231	0.21	<0.001
HDL-cholesterol^c^ (mmol/L)	−0.01	0.831	0.23	<0.001
LDL-cholesterol^c^ (mmol/L)	−0.05	0.176	0.19	<0.001
Fasting glucose^c^ (mmol/L)	−0.01	0.742	0.02	0.573
Systolic blood pressure^c^ (mm Hg)	−0.02	0.482	0.13	<0.001
Diastolic blood pressure^c^ (mm Hg)	−0.04	0.215	0.13	<0.001

Metabolic syndrome (Mets) (Yes (ref. No))	−0.05	0.143	0.08	0.013
*N* of MetS components	−0.04	0.198	0.11	0.001

*N* of chronic diseases	0.01	0.713	0.00	0.988

8-OHdG and F2-isoprostanes were logged transformed for analysis. All analyses were adjusted for research site. Results are reported as standardized regression coefficients. ^a^Analyses on 8-OHdG were adjusted for age, sex, cotinine, and fasting. ^b^Analyses on F2-isoprostanes were adjusted for age, sex, education, cotinine, alcohol use, supplement use, season of sample collection, and fasting.

^c^Triglycerides, HDL-cholesterol, LDL-cholesterol, fasting glucose, systolic and diastolic blood pressure were all adjusted for use of lipid, cholesterol, and glucose or blood pressure lowering medication. HDL: high-density lipoprotein; LDL: low-density lipoprotein; *N*: number; MetS: metabolic syndrome.
